# Genetic Factors in Nonsyndromic Orofacial Clefts

**DOI:** 10.1055/s-0041-1722951

**Published:** 2021-02-12

**Authors:** Mahamad Irfanulla Khan, Prashanth CS, Narasimha Murthy Srinath

**Affiliations:** 1 Department of Orthodontics & Dentofacial Orthopedics, The Oxford Dental College, Bangalore, Karnataka, India; 2 Department of Orthodontics & Dentofacial Orthopedics, DAPM R.V. Dental College, Bangalore, Karnataka, India; 3 Department of Oral & Maxillofacial Surgery, Krishnadevaraya College of Dental Sciences, Bangalore, Karnataka, India

**Keywords:** orofacial clefts, nonsyndromic, genetics, gene mutation, genome-wide association study, linkage analysis

## Abstract

Orofacial clefts (OFCs) are the most common congenital birth defects in humans and immediately recognized at birth. The etiology remains complex and poorly understood and seems to result from multiple genetic and environmental factors along with gene–environment interactions. It can be classified into syndromic (30%) and nonsyndromic (70%) clefts. Nonsyndromic OFCs include clefts without any additional physical or cognitive deficits. Recently, various genetic approaches, such as genome-wide association studies (GWAS), candidate gene association studies, and linkage analysis, have identified multiple genes involved in the etiology of OFCs.

This article provides an insight into the multiple genes involved in the etiology of OFCs. Identification of specific genetic causes of clefts helps in a better understanding of the molecular pathogenesis of OFC. In the near future, it helps to provide a more accurate diagnosis, genetic counseling, personalized medicine for better clinical care, and prevention of OFCs.

## Introduction

Orofacial clefts (OFC) are the most common congenital malformations in the orofacial region causing a significant personal and social encumbrance. 1 2 3 The etiology remains complex involving multiple genetic and environmental factors along with gene–environment interactions. 4 5 6 7 Children born with clefts may have difficulties in sucking, speech, hearing, and unaesthetic facial features due to the anatomical deformities. 8 9 OFCs generally require surgical reconstruction of the lip and palate at different stages from birth to adulthood, 10 and their rehabilitation involves a multidisciplinary approach such as, pediatric care, speech and hearing therapy, dental and orthodontic treatment, genetic counseling, and other mental health therapy. 11 12

## Epidemiology

The prevalence of OFCs ranges from 1 in 700 to 1,000 newborns worldwide 13 and include cleft lip only (CLO), cleft palate only (CPO), and cleft lip and palate (CLP). 14 They may occur as unilateral or bilateral, complete, or incomplete, and may involve the lip only, the palate only, or both. 15

The prevalence of OFC varies according to geographical location, ethnicity, race, gender and socioeconomic status, 16 17 18 19 Asians have the highest prevalence rate (1:500), the intermediate prevalence in Europeans (1:1000), and lowest in Africans (1:2500). 20 21 22 In India, the incidence of clefts is around 1:800 to 1:1000, and three infants are born with some type of cleft every hour. 23 These differences appear to persist even after migration, suggesting that they are mediated by genetic, rather than environmental factors. 24 Overall, 70% of the OFCs are nonsyndromic (NS) and occur as isolated cases without any additional physical or cognitive deficits. In contrast, 30% of clefts are syndromic and are associated with a few other developmental anomalies. 25

The frequency of occurrence of OFC differs with regard to gender and side of clefting. Cleft lip is more common in males at a 2:1 male to female ratio, whereas a cleft palate is more common in females. 26 Approximately 90% of OFCs are unilateral with primarily left-sided involvement. 27

## Development of Cleft Lip and Palate

The development of lip and palate begins during the fourth week of gestation where migrating neural crest cells combine with mesodermal cells to establish facial primordia which consist of five different processes (medial nasal, lateral nasal, frontonasal, maxillary, and mandibular processes) derived from the first pharyngeal arch. 28 Once facial prominences are formed, the nasal placodes invaginate to form the medial nasal process (MNP) and lateral nasal process (LNP). The maxillary processes initially grow medially, pushing the LNP toward the upper side. During the sixth and seventh weeks of gestation fusion of maxillary processes with each other and also with lateral and MNPs takes place to form upper lip and primary palate. 29 Defect in fusion or failure in the growth of these processes results in clefts involving the upper lip, alveolus, and/ or primary palate.

The secondary palate begins to develop in the seventh week of embryogenesis; the maxillary processes initially outgrow as palatal shelves which move toward each other vertically. After proper growth they settle at a horizontal position above the tongue which entails much extracellular remodeling. 30 31 The palatal shelves then fuse in the midline both anteriorly and posteriorly like a zipper that forms a midline epithelial seam (MES). The disintegration of MES is required to maintain palatal confluency, which may involve apoptosis, epithelial-mesenchymal transition (EMT), and cell migration. 32 Effective fusion of the secondary palate results in complete separation of the oral and nasal cavities. Cleft palate can result from failure at any of the steps, including palatal shelves elevation, cell migration, or fusion.

In summary, a variety of cellular mechanisms such as cell proliferation, cell migration, cell growth, cell fusion, apoptosis, EMT, and extracellular remodeling are involved in a coordinated manner during the development of lip and palate. Therefore, disruption in the gene/s involved in these processes during lip and palate development may lead to an OFC.

## Glimpse into the History of Genetic Etiology of Orofacial Clefts

Although the familiarity of OFC has long been noted, Fogh-Andersen was the first to provide the evidence for genetic factors contributing to the etiology of CLP from family-based studies where it was observed that the siblings of CLP patients had an increased frequency of cleft lip with or without cleft palate. 33 This observation was further confirmed by studies on the familial distribution of congenital clefts of the lip and palate, 34 and Dr. Clark Fraser published a review paper highlighting the conclusions of a workshop on CLP sponsored by the National of Institutes of Health of the United States, and he mentioned the etiology is indeed multifactorial. 35 Later, more evidence in favor of contribution of genetic factors to the etiology of CLP accrued from segregation analysis 36 and twin studies 37 38 where the monozygotic twins showed high prevalence rate (40%) than the dizygotic twins (4%).

## Role of Genetic Factors in the Etiology of Orofacial Clefts

The etiology of OFCs involves genetic factors, environmental influences, and gene–environment interactions, all contributing to its susceptibility. Scientific literature evidence suggests that environmental factors such as maternal tobacco smoking and alcohol consumption, antiepileptic medications, maternal folate deficiency, infections, consanguinity, and geographical location are risk factors for NS cleft lip and palate (NSCLP). 39 40 41 42

Advances in genetics and molecular biology techniques have discovered multiple genes and loci associated with CLP. This article provides an overview of the genes implicated in the etiology of NSCLP. Identification of specific genetic variation contributing to NSCLP has led to an increase in our understanding of the molecular pathogenesis of OFC.

## Genes Involved in the Etiology of Nonsyndromic Orofacial Clefts

### Special AT-Rich Sequence-Binding Protein

Special AT-rich sequence-binding protein (SATB2) is a DNA binding protein which binds with nuclear matrix attachment regions. It is involved in transcription regulation and chromatin remodeling process. In an animal study, mouse SATB2 is strongly expressed in the developing cleft palate and is similar to the human SATB2 protein. 43 The identification of SATB2 gene responsible for the craniofacial dysmorphologies associated with deletions and translocations at 2q32-q33, only one region of the genome has been significantly associated with the development of isolated cleft palate. 44 Glass syndrome characterized by cleft palate, gum hyperplasia, slight micrognathia, generalized osteoporosis, and mental retardation reported from Thai patient, 45 was caused by *SATB2* gene mutation. Recently, using salivary miRNAs showed that the *SATB2* genes are involved in the development of the cleft palate and lip development. 46 Table 1 provides the list of genes involved in the etiology of NS OFC in humans.

**Table 1:** Genes involved in the etiology of nonsyndromic orofacial clefts in humans

Gene	Gene symbol	Loci	OMIM	Evidence	References
Special AT-rich sequence-binding protein	*SATB2*	2q33.1	608148	M	43 46
B-cell leukemia/lymphoma 3	*BCL3*	19q13.32	109560	LD, L	47 49
Distal-less homeobox 4	*DLX4*	17q21.33	601911	M	53 55 56
Paired box gene 9	*PAX9*	14q13.3	167416	L, GWAS	6 60 62 64
Netrin 1	*NTN1*	17p13.1	601614	GWAS	70 71
T-box transcription factor 22	*TBX22*	Xq21.1	300307	M, LD	75 77 79
Poliovirus receptor like-1	*PVRL1*	11q23.3	600644	M, LD,GWAS	81 83 85
Cleft lip and palate associated transmembrane protein 1	*CLPTM1*	19q13.32	604783	M, L, LD	89 90
MAF bZIP transcription factor B	*MAFB*	20q12	608968	GWAS	98 101
Fibroblast growth factor receptor 1	*FGFR1*	8p11.23	136350	M, D	102 103
Transcription factor AP2-α	*TFAP2A*	6p24.3	107580	D, L	104
S-glutathione transferase T1	*GSTT1*	22q11.2	600436	LD	108 109
Receptor-like tyrosine kinase	*RYK*	3q22.2	600524	M	110 111
Gamma-aminobutyric acid receptor, Beta-3	*GABRB3*	15q12	137192	GWAS	112
ATP-binding cassette, subfamily A, member 4	*ABCA4*	1p22.1	601691	GWAS	115 117 118

Abbreviations: D, deletions; GWAS, genome-wide association studies; L, linkage; LD, linkage disequilibrium; M, mutations; OMIM, Online Mendelian Inheritance in Man.

### B-Cell Leukemia/Lymphoma 3

B-cell leukemia/lymphoma 3 (BCL3) is a proto-oncogene, acts as a transcriptional co-activator through NF-kappa-B target genes and located on 19q13.2. *BCL3* gene has also shown a strong association with NS orofacial clefts (NSOCs). 47 A case-parent trio study showed BCL3 influence risk of CL/P through a parent-of-origin effect with the excess maternal transmission. 48 Several studies in different populations have implicated the role of BCL3 in development of NSCL/P. It was found that BCL3 contributes to the regulation of cell proliferation, and cell cycle regulation can cause a disturbance in facial formation. 49 A study also reported BCL3 contribution in angiogenesis-related genes in the etiology of CLP. 50 However, no association of *BCL3* gene with NSCLP was found in multigenerational families of Indian population. 51

### Distal-Less Homeobox 4

Distal-Less Homeobox 4 (DLX4) belongs to *DLX* gene family containing a homeobox transcription factor which plays an important role in craniofacial development and palatogenesis. It is located on chromosome 17q21.33 and causes orofacial cleft 15 (OFC15) and cleft lip/palate. In an animal study, the *DLX* genes caused cleft palate showing the importance of these genes in craniofacial morphogenesis. 52 Whole-exome sequencing study in a Hispanic mother and son with bilateral CLP confirmed the DLX4 as a potential cause of oral clefts. 53 54 55 Recently, a study showed that none of the distal-less 4 (DLX4) gene SNPs were associated with NSOCs, so it should be interpreted with a caution in the etiology of nonsyndromic orofacial clefts. 56

### Paired Box Gene 9

Paired Box Gene 9 ( *PAX9* ) is a member of the paired box (PAX) family of transcription factors and contains a paired box domain, an octapeptide, and a paired-type homeodomain. It plays a critical role during neural crest and fetal development. PAX9 is located on chromosome 14q13.3, consists of five exons, and associated with the formation of the teeth and palate. 57 58 Tooth agenesis and the formation of a cleft palate in PAX9-deficient mice have been reported. 59

Schuffenhauer et al, first reported the role of PAX9 in a patient presented with bilateral CLP. 60 A linkage analysis of *PAX9* gene in two large families and four unrelated families showed that the hypodontia primarily involving molars, suggested that the mutant PAX9 protein acquires functional defects in DNA binding, as well as the loss of function of PAX9 resulting in haploinsufficiency during the morphogenesis of the dentition and the subsequent tooth agenesis. 61 62 Several studies identified mutations at PAX9 may increase the risk of NS cleft lip with or without palate. 63 64 65 66

A combined genome-wide association study (GWAS) used unmixed NS CPO and NS CLO subtypes suggested that PAX9 is a strong genetic factor for NS CPO in the Chinese population. Mutation analysis of PAX9 SNPs rs12885612 and rs12881248 revealed that *PAX9* is a promising susceptible gene for NS CLO in Western Han Chinese population. 5 6

### Netrin 1 (NTN1)

The netrin 1 (OMIM: 601614) located on chromosome 17p13.1 is a family of laminin-related secreted proteins. Its functions include axon guidance and cell migration in the central nervous system, angiogenesis, and semicircular canal formation.

NTN1 is expressed at high levels in cells that will come together to form a fusion plate, a prerequisite for the formation of semicircular canals. In netrin 1 mutant mice, fusion plate formation is severely affected, and it stimulates proliferation of the periotic mesenchymal cells which then push the epithelial cell walls together to form the fusion plate. 67 68 It affects the development of the craniofacial region and has been shown to play a vital role in regulating cell migration during embryogenesis, and it is also expressed in the medial edges and oral sides of the palatal shelves. 69

A GWAS included 1,409 case-parent trios by several research groups with samples of Asian or European ancestry from Europe, the United States, China, and the Philippines found NTN1 role in the etiology of NSCLP. 70 A case–control study of NTN1, identified SNP (rs9788972) as a risk locus for NSOCs susceptibility in a northern Chinese population. 71

### T-Box Transcription Factor 22

The *T-box 22* (TBX22) gene encodes transcription factors involved in the regulation of developmental processes and plays a major role in human palatogenesis. It contains eight coding exons. TBX22 cause X-linked cleft palate and is characterized by isolated cleft palate and ankyloglossia. 72 It is expressed in the developing palatal shelves and at the base of the tongue prior to elevation to a horizontal position above the tongue. 73

Genome-wide linkage analysis showed the association of TBX22 role in the development of NSCLP. 74 In addition, mutations in TBX22 were found in individuals with isolated CPO. 75 76 77 It plays a significant role in tooth and upper lip development and causes hypodontia and cleft lip. 78 DNA methylation study suggests that cleft palate-susceptible gene Tbx22 is associated with gene expression and might be responsible for the developmental failure of palatal fusion, eventually resulting in the formation of cleft palate. 79

### Poliovirus Receptor-Like 1

Poliovirus receptor-like 1 (PVRL1), also known as NECTIN1 (nectin cell adhesion molecule 1) belongs to the nectin subfamily of immunoglobulin-like adhesion molecules that involve cell–cell adhesion. It plays a vital role in the organization of adherens junctions and tight junctions in epithelial and endothelial cells. 80 During the developmental process, the palatal shelves and palatal epithelium come in close contact and fuse together. PVRL1 plays a major role in these developments during palatogenesis and genetic variations reported to have a significant relationship with CLP. Diseases associated with PVRL1 include cleft lip/palate-ectodermal dysplasia syndrome (CLPED) and herpes simplex.

In animal experiments, PVRL1 expressed at the medial edge epithelium of the palatal shelves and the skin surface epithelium locations that corresponded to the clinical phenotypes of CLPED and in humans, mutations of the *PVRL1* gene resulting CLPED in families from Israel and Brazil. 81 Interestingly, heterozygous mutation of PVRL1 (W185X) associated with NSCLP in northern Venezuela 82 83 and two novel variants of the *PVRL1* gene were identified in Turkish NSCLP patients. 84 In an experimental animal studies, PVRL1 expressed at the medial edge epithelium of the palatal shelves and the skin surface epithelium locations that corresponded to the clinical phenotypes of CLPED and in humans, mutations of the PVRL1 genes caused CLPED in Israel and Brazilian population. 85 86 87 88

### Cleft Lip and Palate-Associated Transmembrane Protein 1

Cleft lip and palate-associated transmembrane protein 1 (CLPTM1) is a multipass transmembrane protein that regulates GABA-A receptors (e.g., GABRA1) and modulates inhibitory synaptic strength. It is located on chromosome 19q13.3 and plays a role in T-cell development. Mutation of *CLPTM1* genes suggested that a regulatory element in this gene region get affected and which is responsible for the development of orofacial clefts. 89 90 However, some studies contradict the role of CLPTM1 in the etiology of NSCL/P as no evidence of an association with oral clefts was found among the SNPs of CLPTM1 selected for testing in Japanese and Irish population. 91 92

### MAF bZIP Transcription Factor B

The MAF bZIP Transcription Factor B *(MAFB)* gene encodes a basic leucine zipper (bZIP) transcription factor that plays an important role in the regulation of lineage-specific hematopoiesis. It is located on chromosome 20q11.2, consists of a single exon and spans approximately 3 kb. 93 In a mouse study, MAFB was expressed in the palatal shelves and the medial edge epithelia (or MEE) during palatal fusion. 94 Mutations in the *MAFB* gene reported causing multicentric carpotarsal osteolysis syndrome and Duane retraction syndrome 3 with or without deafness. 95 96

Several GWAS in European and Asian populations identified the role of MAFB in NS CLP. 97 98 Different systematic review and meta-analyses confirmed that the *MAFB* gene SNP (rs13041247) is associated with NSCL/P risk in different population; however, this association is not significant in East Asian or Caucasian populations, 99 100 whereas, the SNPs rs17820943 and rs6072081 of MAFB found to be associated with NSCLP in an East Asian population. 101

### Other Candidate Genes

A variety of genetic approaches have identified several genes located on different chromosomes, contributing to etiology of NSCLP. Advances in genetics and molecular biology techniques have led the way to the discovery of genetic variation involved in NSCLP. Genes such as FGFR1, TFAP2A, GSTT1, receptor-like tyrosine kinase (RYK), and ABCA4 have also been found to be associated with NSCLP.

Riley et al assessed the genes involved in the fibroblast growth factor (FGF) signaling pathway and identified the functional impairment in the *FGFR1* gene in NSCLP families and suggested that the FGF signaling pathway may contribute to as much as 3 to 5% of NS cleft lip or palate. 102 Xu et al reported mutations of the *FGFR1* gene in Chinese Kallmann syndrome males with cleft lip/palate. 103 TFAP2A located on 6p24 region has been associated with orofacial clefting. Davies et al reported a patient with cleft palate, microretrognathia, frontal bossing, hypertelorism, flat, broad nasal bridge, low set ears, and developmental delay. 104 However, it was found that there was no significant association between TFAP2A and NSCLP in this northern Chinese 105 and Indian population. 106 S-glutathione transferase T1 (GSTT1) play an important role in the detoxification and secretion of smoking byproducts and deficiency of this enzyme may cause a greater risk of NSCLP if the individuals were exposed to smoking byproducts during pregnancy. 107 108 Hozyasz et al suggested that homozygous deletion of GSTT1 in mother genome might increase the risk of having a child with NSCLP. 109

Further, mutations in the *RYK* gene were also found to be associated with orofacial clefting. 110 111 Significant linkage disequilibrium between Gamma-aminobutyric acid receptor, Beta-3 (GABRB3), and CLP was reported by Scapoli et al and this finding in humans is in agreement with previously reported data obtained with the murine model. 112 In addition, Baroni et al and Carter et al reported the association of GABRB3 with oral clefts in different populations. 113 114 ATP-binding cassette, subfamily A, member 4 (ABCA4) encodes an ATP-binding cassette transporter. Several linkages and GWAS showed the role of ABCA4 in NSCLP with stronger evidence among Asian samples. ABCA4 is known to cause the autosomal-recessive retinal degenerative disease Stargardt's disease. A GWAS and a case-parent trio approach by Beaty et al identified *ABCA4* gene association with NSCLP 115 116 and several other studies also reported a potential role of ABCA4 in the etiology of CL/P in Brazilian and northern Chinese Han population. 117 118

## Conclusion

Genetic studies provide insight into the multiple genes involved in the etiology of OFCs. Identification of specific genetic causes of clefts helps for better understanding of the molecular pathogenesis of OFC. In the near future, it helps to provide a more accurate diagnosis, genetic counseling, personalized medicine for better clinical care and prevention of OFCs.

## References

[1] Wong F K Hagg U An update on the aetiology of orofacial clefts Hong Kong Med J 2004 10 05 331 336 15479962

[2] Mossey P Epidemiology underpinning research in the aetiology of orofacial clefts Orthod Craniofac Res 2007 10 03 114 120 17651127 10.1111/j.1601-6343.2007.00398.x

[3] Rahimov F Jugessur A Murray J C Genetics of nonsyndromic orofacial clefts Cleft Palate Craniofac J 2012 49 01 73 91 21545302 10.1597/10-178 PMC3437188

[4] Worley M L Patel K G Kilpatrick L A Cleft lip and palate Clin Perinatol 2018 45 04 661 678 30396411 10.1016/j.clp.2018.07.006

[5] Huang L Jia Z Shi Y Genetic factors define CPO and CLO subtypes of nonsyndromicorofacial cleft PLoS Genet 2019 15 10 e1008357 31609978 10.1371/journal.pgen.1008357 PMC6812857

[6] Yang C W Shi J Y Yin B Shi B Jia Z L Mutation at paired box gene 9 is associated with non-syndromic cleft lip only from Western Han Chinese population Arch Oral Biol 2020 117 104829 32679372 10.1016/j.archoralbio.2020.104829

[7] Martinelli M Palmieri A Carinci F Scapoli L Non-syndromic cleft palate: an overview on human genetic and environmental risk factors Front Cell Dev Biol 2020 8 592271 33195260 10.3389/fcell.2020.592271 PMC7606870

[8] Jugessur A Farlie P G Kilpatrick N The genetics of isolated orofacial clefts: from genotypes to subphenotypes Oral Dis 2009 15 07 437 453 19583827 10.1111/j.1601-0825.2009.01577.x

[9] do Rego Borges A Sá J Hoshi R Genetic risk factors for nonsyndromic cleft lip with or without cleft palate in a Brazilian population with high African ancestry Am J Med Genet A 2015 167A 10 2344 2349 26198054 10.1002/ajmg.a.37181

[10] Saad A N Parina R P Tokin C Chang D C Gosman A Incidence of oral clefts among different ethnicities in the state of California Ann Plast Surg 2014 72 01 S81 S83 24691321 10.1097/SAP.0000000000000164

[11] Ghazali N Rahman N A Kannan T P Jaafar S Screening of transforming growth factor Beta 3 and Jagged2 genes in the Malay population with nonsyndromic cleft lip with or without cleft palate Cleft Palate Craniofac J 2015 52 04 e88 e94 26151095 10.1597/14-024

[12] Hoffman-Andrews L Tarnowski J M Lee S Characteristics of orofacial clefting in Hawai'i Hawaii J Health Soc Welf 2019 78 08 258 261 31463475 PMC6695337

[13] Dixon M J Marazita M L Beaty T H Murray J C Cleft lip and palate: understanding genetic and environmental influences Nat Rev Genet 2011 12 03 167 178 21331089 10.1038/nrg2933 PMC3086810

[14] Leslie E J Marazita M L Genetics of cleft lip and cleft palate Am J Med Genet C Semin Med Genet 2013 163C 04 246 258 24124047 10.1002/ajmg.c.31381 PMC3925974

[15] Neela P K Gosla S R Husain A Mohan V *CRISPLD2* gene polymorphisms with nonsyndromic cleft lip palate in Indian population Glob Med Genet 2020 7 01 22 25 32879920 10.1055/s-0040-1713166 PMC7410092

[16] Hagberg C Larson O Milerad J Incidence of cleft lip and palate and risks of additional malformations Cleft Palate Craniofac J 1998 35 01 40 45 9482222 10.1597/1545-1569_1998_035_0040_ioclap_2.3.co_2

[17] Ogle O E Incidence of cleft lip and palate in a newborn Zairian sample Cleft Palate Craniofac J 1993 30 02 250 251 8452851

[18] Wyszynski D F Wu T Use of US birth certificate data to estimate the risk of maternal cigarette smoking for oral clefting Cleft Palate Craniofac J 2002 39 02 188 192 11879077 10.1597/1545-1569_2002_039_0188_uousbc_2.0.co_2

[19] National Birth Defects Prevention Study Yang J Carmichael S L Canfield M Song J Shaw G M Socioeconomic status in relation to selected birth defects in a large multicentered US case-control study Am J Epidemiol 2008 167 02 145 154 17947220 10.1093/aje/kwm283

[20] Croen L A Shaw G M Wasserman C R Tolarová M M Racial and ethnic variations in the prevalence of orofacial clefts in California, 1983-1992 Am J Med Genet 1998 79 01 42 47 9738868 10.1002/(sici)1096-8628(19980827)79:1<42::aid-ajmg11>3.0.co;2-m

[21] Mossey P A Little J Munger R G Dixon M J Shaw W C Cleft lip and palate Lancet 2009 374(9703):1773 1785 19747722 10.1016/S0140-6736(09)60695-4

[22] National Birth Defects Prevention Network Parker S E Mai C T Canfield M A Updated National Birth Prevalence estimates for selected birth defects in the United States, 2004-2006 Birth Defects Res A Clin Mol Teratol 2010 88 12 1008 1016 20878909 10.1002/bdra.20735

[23] Neela P K Gosla S R Husain A Mohan V Thumoju S Rajeshwari B V Analysis of single nucleotide polymorphisms on locus 13q33.1–34 in multigenerational families of cleft lip palate using mass arrayIndones Biomed J. 2020; In press

[24] Bender P L Genetics of cleft lip and palate J Pediatr Nurs 2000 15 04 242 249 10969497 10.1053/jpdn.2000.8148

[25] Neela P K Gosla S R Husain A Mohan V Thumoju S Bv R Association of MAPK4 and SOX1-OT gene polymorphisms with cleft lip palate in multiplex families: a genetic study J Dent Res Dent Clin Dent Prospect 2020 14 02 93 96 10.34172/joddd.2020.021 PMC7464228 32908649

[26] Wyszynski D F Wu T Prenatal and perinatal factors associated with isolated oral clefting Cleft Palate Craniofac J 2002 39 03 370 375 12019016 10.1597/1545-1569_2002_039_0370_papfaw_2.0.co_2

[27] Cobourne M T Cleft Lip and Palate. Epidemiology, Aetiology and Treatment. Front Oral Biol London Karger 2012 60 70

[28] Singh I Pal G P Human Embryology 7th ed. Delhi Macmillan India Ltd 2001 130 131

[29] Jiang R Bush J O Lidral A C Development of the upper lip: morphogenetic and molecular mechanisms Dev Dyn 2006 235 05 1152 1166 16292776 10.1002/dvdy.20646 PMC2562450

[30] Stanier P Moore G E Genetics of cleft lip and palate: syndromic genes contribute to the incidence of non-syndromic clefts Hum Mol Genet 2004 13(Spec No 1):R73 R81 14722155 10.1093/hmg/ddh052

[31] Gritli-Linde A Molecular control of secondary palate development Dev Biol 2007 301 02 309 326 16942766 10.1016/j.ydbio.2006.07.042

[32] Bush J O Jiang R Palatogenesis: morphogenetic and molecular mechanisms of secondary palate development Development 2012 139 02 231 243 22186724 10.1242/dev.067082 PMC3243091

[33] Fogh Andersen P Inheritance of harelip and cleft palate Copenhagen Busck 1942

[34] Fraser F C Baxter H The familial distribution of congenital clefts of the lip and palate; a preliminary report Am J Surg 1954 87 05 656 659 13148441 10.1016/0002-9610(54)90161-0

[35] Fraser F C The genetics of cleft lip and cleft palate Am J Hum Genet 1970 22 03 336 352 4910698 PMC1706547

[36] Marazita M L Spence M A Melnick M Genetic analysis of cleft lip with or without cleft palate in Danish kindreds Am J Med Genet 1984 19 01 9 18 6496575 10.1002/ajmg.1320190104

[37] Mitchell L E Mode of inheritance of oral clefts New York, NY Oxford University Press 2002 234 239

[38] Grosen D Bille C Pedersen J K Skytthe A Murray J C Christensen K Recurrence risk for offspring of twins discordant for oral cleft: a population-based cohort study of the Danish 1936-2004 cleft twin cohort Am J Med Genet A 2010 152A 10 2468 2474 20799319 10.1002/ajmg.a.33608 PMC2946514

[39] Chung K C Kowalski C P Kim H M Buchman S R Maternal cigarette smoking during pregnancy and the risk of having a child with cleft lip/palate Plast Reconstr Surg 2000 105 02 485 491 10697150 10.1097/00006534-200002000-00001

[40] Bhaskar L V Murthy J Venkatesh Babu G Polymorphisms in genes involved in folate metabolism and orofacial clefts Arch Oral Biol 2011 56 08 723 737 21310392 10.1016/j.archoralbio.2011.01.007

[41] Sabbagh H J Innes N P Sallout B I Birth prevalence of non-syndromic orofacial clefts in Saudi Arabia and the effects of parental consanguinity Saudi Med J 2015 36 09 1076 1083 26318465 10.15537/smj.2015.9.11823 PMC4613632

[42] Neela P K Reddy S G Husain A Mohan V Association of cleft lip and/or palate in people born to consanguineous parents: a 13-year retrospective study from a very high volume cleft center J Cleft Lip Palate Craniofacial Anomalies 2019 6 33 37

[43] FitzPatrick D R Carr I M McLaren L Identification of SATB2 as the cleft palate gene on 2q32-q33 Hum Mol Genet 2003 12 19 2491 2501 12915443 10.1093/hmg/ddg248

[44] Britanova O Depew M J Schwark M SATB2 haploinsufficiency phenocopies 2q32-q33 deletions, whereas loss suggests a fundamental role in the coordination of jaw development Am J Hum Genet 2006 79 04 668 678 16960803 10.1086/508214 PMC1592575

[45] Leoyklang P Suphapeetiporn K Siriwan P Heterozygous nonsense mutation SATB2 associated with cleft palate, osteoporosis, and cognitive defects Hum Mutat 2007 28 07 732 738 17377962 10.1002/humu.20515

[46] Grassia V Lombardi A Kawasaki H Salivary microRNAs as new molecular markers in cleft lip and palate: a new frontier in molecular medicine Oncotarget 2018 9 27 18929 18938 29721173 10.18632/oncotarget.24838 PMC5922367

[47] Martinelli M Scapoli L Pezzetti F Suggestive linkage between markers on chromosome 19q13.2 and nonsyndromic orofacial cleft malformation Genomics 1998 51 02 177 181 9722939 10.1006/geno.1998.5384

[48] Park B Y Sull J W Park J Y Jee S H Beaty T H Differential parental transmission of markers in BCL3 among Korean cleft case-parent trios J Prev Med Public Health 2009 42 01 1 4 19229118 10.3961/jpmph.2009.42.1.1 PMC2882880

[49] Lace B Kempa I Klovins J *BCL3* gene role in facial morphology Birth Defects Res A Clin Mol Teratol 2012 94 11 918 924 23115114 10.1002/bdra.23085

[50] François-Fiquet C Poli-Merol M L Nguyen P Landais E Gaillard D Doco-Fenzy M Role of angiogenesis-related genes in cleft lip/palate: review of the literature Int J Pediatr Otorhinolaryngol 2014 78 10 1579 1585 25176321 10.1016/j.ijporl.2014.08.001

[51] Neela P K Gosla S R Husain A Mohan V Thumoju S Rajeshwari B V Association of nucleotide variants of *GRHL3, IRF6, NAT2, SDC2, BCL3* and *PVRL1* genes with non-syndromic cleft lip with/without cleft palate in multigenerational families: a retrospective study Contemp Clin Dent 2020; In press 10.4103/ccd.ccd_329_20 PMC8237814 34220153

[52] Talbot J C Johnson S L Kimmel C B hand2 and Dlx genes specify dorsal, intermediate and ventral domains within zebrafish pharyngeal arches Development 2010 137 15 2507 2517 20573696 10.1242/dev.049700 PMC2927700

[53] Murthi P Said J M Doherty V L Homeobox gene DLX4 expression is increased in idiopathic human fetal growth restriction Mol Hum Reprod 2006 12 12 763 769 17062780 10.1093/molehr/gal087

[54] Zhang L Yang M Gan L DLX4 upregulates TWIST and enhances tumor migration, invasion and metastasis Int J Biol Sci 2012 8 08 1178 1187 23091415 10.7150/ijbs.4458 PMC3477687

[55] Wu D Mandal S Choi A DLX4 is associated with orofacial clefting and abnormal jaw development Hum Mol Genet 2015 24 15 4340 4352 25954033 10.1093/hmg/ddv167 PMC4492397

[56] He M Bian Z Association between DLX4 polymorphisms and nonsyndromicorofacial clefts in a Chinese Han population Cleft Palate Craniofac J 2019 56 03 357 362 29738288 10.1177/1055665618775723

[57] Bonczek O Balcar V J Šerý O *PAX9* gene mutations and tooth agenesis: a review Clin Genet 2017 92 05 467 476 28155232 10.1111/cge.12986

[58] Li R Chen Z Yu Q Weng M Chen Z The function and regulatory network of *PAX9* gene in palate development J Dent Res 2019 98 03 277 287 30583699 10.1177/0022034518811861

[59] Peters H Neubüser A Kratochwil K Balling R *PAX9* -deficient mice lack pharyngeal pouch derivatives and teeth and exhibit craniofacial and limb abnormalities Genes Dev 1998 12 17 2735 2747 9732271 10.1101/gad.12.17.2735 PMC317134

[60] Schuffenhauer S Leifheit H J Lichtner P Peters H Murken J Emmerich P De novo deletion (14)(q11.2q13) including PAX9: clinical and molecular findings J Med Genet 1999 36 03 233 236 10204852 PMC1734334

[61] Das P Hai M Elcock C Novel missense mutations and a 288-bp exonic insertion in PAX9 in families with autosomal dominant hypodontia Am J Med Genet A 2003 118A 01 35 42 12605438 10.1002/ajmg.a.10011 PMC6156786

[62] Zhao J L Chen Y X Bao L Xia Q J Wu T J Zhou L Novel mutations of *PAX9* gene in Chinese patients with oligodontia Zhonghua Kou Qiang Yi Xue Za Zhi 2005 40 04 266 270 16191360

[63] Sull J W Liang K Y Hetmanski J B Maternal transmission effects of the PAX genes among cleft case-parent trios from four populations Eur J Hum Genet 2009 17 06 831 839 19142206 10.1038/ejhg.2008.250 PMC2760446

[64] Nakatomi M Wang X P Key D Genetic interactions between PAX9 and Msx1 regulate lip development and several stages of tooth morphogenesis Dev Biol 2010 340 02 438 449 20123092 10.1016/j.ydbio.2010.01.031

[65] Song T Wu D Wang Y Li H Yin N Zhao Z SNPs and interaction analyses of IRF6, MSX1 and *PAX9* genes in patients with nonsyndromic cleft lip with or without palate Mol Med Rep 2013 8 04 1228 1234 23921572 10.3892/mmr.2013.1617

[66] de Araujo T K Secolin R Félix T M A multicentric association study between 39 genes and nonsyndromic cleft lip and palate in a Brazilian population J Craniomaxillofac Surg 2016 44 01 16 20 26602496 10.1016/j.jcms.2015.07.026

[67] Serafini T Colamarino S A Leonardo E D Netrin-1 is required for commissural axon guidance in the developing vertebrate nervous system Cell 1996 87 06 1001 1014 8978605 10.1016/s0092-8674(00)81795-x

[68] Salminen M Meyer B I Bober E Gruss P Netrin 1 is required for semicircular canal formation in the mouse inner ear Development 2000 127 01 13 22 10654596 10.1242/dev.127.1.13

[69] Srinivasan K Strickland P Valdes A Shin G C Hinck L Netrin-1/neogenin interaction stabilizes multipotent progenitor cap cells during mammary gland morphogenesis Dev Cell 2003 4 03 371 382 12636918 10.1016/s1534-5807(03)00054-6

[70] Leslie E J Taub M A Liu H Identification of functional variants for cleft lip with or without cleft palate in or near PAX7, FGFR2, and NOG by targeted sequencing of GWAS loci Am J Hum Genet 2015 96 03 397 411 25704602 10.1016/j.ajhg.2015.01.004 PMC4375420

[71] Guo Q Li D Meng X Association between *PAX7* and *NTN1* gene polymorphisms and nonsyndromicorofacial clefts in a northern Chinese population Medicine (Baltimore) 2017 96 19 (e6724) 10.1097/MD.0000000000006724 PMC5428583 28489749

[72] Braybrook C Doudney K Marçano A C The T-box transcription factor gene TBX22 is mutated in X-linked cleft palate and ankyloglossia Nat Genet 2001 29 02 179 183 11559848 10.1038/ng730

[73] Braybrook C Lisgo S Doudney K Craniofacial expression of human and murine TBX22 correlates with the cleft palate and ankyloglossia phenotype observed in CPX patients Hum Mol Genet 2002 11 22 2793 2804 12374769 10.1093/hmg/11.22.2793

[74] Prescott N J Lees M M Winter R M Malcolm S Identification of susceptibility loci for nonsyndromic cleft lip with or without cleft palate in a two stage genome scan of affected sib-pairs Hum Genet 2000 106 03 345 350 10798365 10.1007/s004390051048

[75] Marçano A C Doudney K Braybrook C TBX22 mutations are a frequent cause of cleft palate J Med Genet 2004 41 01 68 74 14729838 10.1136/jmg.2003.010868 PMC1757272

[76] Suphapeetiporn K Tongkobpetch S Siriwan P Shotelersuk V TBX22 mutations are a frequent cause of non-syndromic cleft palate in the Thai population Clin Genet 2007 72 05 478 483 17868388 10.1111/j.1399-0004.2007.00891.x

[77] Dai J Xu C Wang G Novel *TBX22* mutations in Chinese nonsyndromic cleft lip/palate families J Genet 2018 97 02 411 417 29932061

[78] Kantaputra P N Paramee M Kaewkhampa A Cleft lip with cleft palate, ankyloglossia, and hypodontia are associated with TBX22 mutations J Dent Res 2011 90 04 450 455 21248356 10.1177/0022034510391052

[79] Shu X Dong Z Cheng L Shu S DNA hypermethylation of Fgf16 and Tbx22 associated with cleft palate during palatal fusion J Appl Oral Sci 2019 27 e20180649 31596367 10.1590/1678-7757-2018-0649 PMC6768118

[80] Takahashi K Nakanishi H Miyahara M Nectin/PRR: an immunoglobulin-like cell adhesion molecule recruited to cadherin-based adherens junctions through interaction with Afadin, a PDZ domain-containing protein J Cell Biol 1999 145 03 539 549 10225955 10.1083/jcb.145.3.539 PMC2185068

[81] Suzuki K Hu D Bustos T Mutations of PVRL1, encoding a cell-cell adhesion molecule/herpesvirus receptor, in cleft lip/palate-ectodermal dysplasia Nat Genet 2000 25 04 427 430 10932188 10.1038/78119

[82] Sözen M A Suzuki K Tolarova M M Bustos T Fernández Iglesias J E Spritz R A Mutation of PVRL1 is associated with sporadic, non-syndromic cleft lip/palate in northern Venezuela Nat Genet 2001 29 02 141 142 11559849 10.1038/ng740

[83] Scapoli L Palmieri A Martinelli M Study of the *PVRL1* gene in Italian nonsyndromic cleft lip patients with or without cleft palate Ann Hum Genet 2006 70(Pt 3):410 413 16674562 10.1111/j.1529-8817.2005.00237.x

[84] Oner D A Tastan H Identification of novel variants in the *PVRL1* gene in patients with nonsyndromic cleft lip with or without cleft palate Genet Test Mol Biomarkers 2016 20 05 269 272 26953873 10.1089/gtmb.2015.0276

[85] Avila J R Jezewski P A Vieira A R PVRL1 variants contribute to non-syndromic cleft lip and palate in multiple populations Am J Med Genet A 2006 140 23 2562 2570 17089422 10.1002/ajmg.a.31367 PMC1885468

[86] Sözen M A Hecht J T Spritz R A Mutation analysis of the *PVRL1* gene in Caucasians with nonsyndromic cleft lip/palate Genet Test Mol Biomarkers 2009 13 05 617 621 19715471 10.1089/gtmb.2009.0052 PMC2953240

[87] Cheng H Q Huang E M Xu M Y Shu S Y Tang S J PVRL1 as a candidate gene for nonsyndromic cleft lip with or without cleft palate: no evidence for the involvement of common or rare variants in southern Han Chinese patients DNA Cell Biol 2012 31 07 1321 1327 22455396 10.1089/dna.2011.1556 PMC3391494

[88] Shu S Y Zhang M J Cheng H Q Mutation analysis of PVRL1 in patients with non-syndromic cleft of the lip and/or palate in Guangdong Genet Mol Res 2015 14 02 3400 3408 25966106 10.4238/2015.April.15.3

[89] Yoshiura K Machida J Daack-Hirsch S Characterization of a novel gene disrupted by a balanced chromosomal translocation t(2;19)(q11.2;q13.3) in a family with cleft lip and palate Genomics 1998 54 02 231 240 9828125 10.1006/geno.1998.5577

[90] Warrington A Vieira A R Christensen K Genetic evidence for the role of loci at 19q13 in cleft lip and palate J Med Genet 2006 43 06 e26 16740910 10.1136/jmg.2005.034785 PMC2564544

[91] Ichikawa E Watanabe A Nakano Y PAX9 and TGFB3 are linked to susceptibility to nonsyndromic cleft lip with or without cleft palate in the Japanese: population-based and family-based candidate gene analyses J Hum Genet 2006 51 01 38 46 16247549 10.1007/s10038-005-0319-8

[92] Carter T C Molloy A M Pangilinan F Testing reported associations of genetic risk factors for oral clefts in a large Irish study population Birth Defects Res A Clin Mol Teratol 2010 88 02 84 93 19937600 10.1002/bdra.20639 PMC3503531

[93] Wang P W Eisenbart J D Cordes S P Barsh G S Stoffel M Le Beau M M Human KRML (MAFB): cDNA cloning, genomic structure, and evaluation as a candidate tumor suppressor gene in myeloid leukemias Genomics 1999 59 03 275 281 10444328 10.1006/geno.1999.5884

[94] Beaty T H Murray J C Marazita M L A genome-wide association study of cleft lip with and without cleft palate identifies risk variants near MAFB and ABCA4 Nat Genet 2010 42 06 525 529 20436469 10.1038/ng.580 PMC2941216

[95] Zankl A Duncan E L Leo P J Multicentric carpotarsal osteolysis is caused by mutations clustering in the amino-terminal transcriptional activation domain of MAFB Am J Hum Genet 2012 90 03 494 501 22387013 10.1016/j.ajhg.2012.01.003 PMC3309183

[96] Park J G Tischfield M A Nugent A A Loss of MAFB function in humans and mice causes Duane syndrome, aberrant extraocular muscle innervation, and inner-ear defects Am J Hum Genet 2016 98 06 1220 1227 27181683 10.1016/j.ajhg.2016.03.023 PMC4908193

[97] Mi N Hao Y Jiao X Association study of single nucleotide polymorphisms of MAFB with non-syndromic cleft lip with or without cleft palate in a population in Heilongjiang Province, northern China Br J Oral Maxillofac Surg 2014 52 08 746 750 24972815 10.1016/j.bjoms.2014.06.003

[98] He Y Huang L Zheng Y Chen J H Tang S Association of single nucleotide polymorphisms at 20q12 with nonsyndromic cleft lip with or without cleft palate in a Southern Chinese Han cohort Mol Genet Genomic Med 2020 8 01 e1028 31713353 10.1002/mgg3.1028 PMC6978266

[99] Imani M M Lopez-Jornet P Pons-Fuster López E Sadeghi M Polymorphic variants of V-Maf musculoaponeurotic fibrosarcoma oncogene homolog B (rs13041247 and rs11696257) and risk of non-syndromic cleft lip/palate: systematic review and meta-analysis Int J Environ Res Public Health 2019 16 15 2792 10.3390/ijerph16152792 PMC6695977 31387249

[100] Huang L Liang X Ou Y Tang S He Y Association between 20q12 rs13041247 polymorphism and risk of nonsyndromic cleft lip with or without cleft palate: a meta-analysis BMC Oral Health 2020 20 01 39 32019513 10.1186/s12903-020-1003-2 PMC7001214

[101] Liang X Huang L Ou Y He Y Tang S Association between MAFB rs17820943 and rs6072081 polymorphism and risk of nonsyndromic cleft lip with or without cleft palate: a meta-analysis Br J Oral Maxillofac Surg 2020 58 09 1065 1072 32646788 10.1016/j.bjoms.2020.05.034

[102] Riley B M Mansilla M A Ma J Impaired FGF signaling contributes to cleft lip and palate Proc Natl Acad Sci U S A 2007 104 11 4512 4517 17360555 10.1073/pnas.0607956104 PMC1810508

[103] Xu H Niu Y Wang T Novel FGFR1 and KISS1R mutations in Chinese Kallmann syndrome males with cleft lip/palate BioMed Res Int 2015 2015 649698 26199944 10.1155/2015/649698 PMC4496468

[104] Davies A F Imaizumi K Mirza G Further evidence for the involvement of human chromosome 6p24 in the aetiology of orofacial clefting J Med Genet 1998 35 10 857 861 9783713 10.1136/jmg.35.10.857 PMC1051465

[105] Shi J Song T Jiao X Qin C Zhou J Single-nucleotide polymorphisms (SNPs) of the IRF6 and TFAP2A in non-syndromic cleft lip with or without cleft palate (NSCLP) in a northern Chinese population Biochem Biophys Res Commun 2011 410 04 732 736 21683068 10.1016/j.bbrc.2011.06.006

[106] Babu Gurramkonda V Syed A H Murthy J Association of *TFAP2A* gene polymorphism with susceptibility to non-syndromic cleft lip with or without palate risk in south Indian population Meta Gene 2016 9 181 184 27617216 10.1016/j.mgene.2016.07.007 PMC5006125

[107] Cobourne M T The complex genetics of cleft lip and palate Eur J Orthod 2004 26 01 7 16 14994877 10.1093/ejo/26.1.7

[108] Shi M Christensen K Weinberg C R Orofacial cleft risk is increased with maternal smoking and specific detoxification-gene variants Am J Hum Genet 2007 80 01 76 90 17160896 10.1086/510518 PMC1785306

[109] Hozyasz K K Mostowska A Surowiec Z Jagodziński P P [Genetic polymorphisms of GSTM1 and GSTT1 in mothers of children with isolated cleft lip with or without cleft palate] Przegl Lek 2005 62 10 1019 1022 16521944

[110] Watanabe A Akita S Tin N T A mutation in RYK is a genetic factor for nonsyndromic cleft lip and palate Cleft Palate Craniofac J 2006 43 03 310 316 16681403 10.1597/04-145.1

[111] Smane L Pilmane M IRF6, RYK, and PAX9 expression in facial tissue of children with cleft palate Int J Morphol 2015 33 647 652

[112] Scapoli L Martinelli M Pezzetti F Linkage disequilibrium between *GABRB3* gene and nonsyndromic familial cleft lip with or without cleft palate Hum Genet 2002 110 01 15 20 11810291 10.1007/s00439-001-0639-5

[113] Baroni T Bellucci C Lilli C Retinoic acid, GABA-ergic, and TGF-beta signaling systems are involved in human cleft palate fibroblast phenotype Mol Med 2006 12(9-10):237 245 17225872 10.2119/2006-00026.Baroni PMC1770008

[114] Vieira A R Howe A Murray J C Studies of γ-aminobutyric acid type A receptor β3 (GABRB3) and glutamic acid decarboxylase 67 (GAD67) with oral clefts Am J Med Genet A 2008 146A 21 2828 2830 18837046 10.1002/ajmg.a.32260 PMC2680820

[115] Fontoura C Silva R M Granjeiro J M Letra A Further evidence of association of the *ABCA4* gene with cleft lip/palate Eur J Oral Sci 2012 120 06 553 557 23167473 10.1111/eos.12001 PMC4438764

[116] Beaty T H Taub M A Scott A F Confirming genes influencing risk to cleft lip with/without cleft palate in a case-parent trio study Hum Genet 2013 132 07 771 781 23512105 10.1007/s00439-013-1283-6 PMC3707506

[117] Mi N Hao Y Jiao X Zheng X Shi J Chen Y A polymorphic marker associated with non-syndromic cleft lip with or without cleft palate in a population in Heilongjiang Province, northern China Arch Oral Biol 2015 60 02 357 361 25499508 10.1016/j.archoralbio.2014.11.009

[118] Peng H H Chang N C Chen K T Nonsynonymous variants in MYH9 and ABCA4 are the most frequent risk loci associated with nonsyndromic orofacial cleft in Taiwanese population BMC Med Genet 2016 17 01 59 27527345 10.1186/s12881-016-0322-2 PMC4986225

[OR2000022-119] National Center for Biotechnology Information (NCBI). Accessed August 20, 2020 at:http://www.ncbi.nih.gov/

[OR2000022-120] Online Mendelian Inheritance in Man (OMIM). Accessed August 20, 2020 at:http://www.ncbi.nlm.nih.gov/omim/

